# Is Experiential Avoidance a Mediating, Moderating, Independent, Overlapping, or Proxy Risk Factor in the Onset, Relapse and Maintenance of Depressive Disorders?

**DOI:** 10.1007/s10608-015-9747-8

**Published:** 2015-12-30

**Authors:** Philip Spinhoven, Jolijn Drost, Mark de Rooij, Albert M. van Hemert, Brenda W. J. H. Penninx

**Affiliations:** Institute of Psychology, Leiden University, Wassenaarseweg 52, 2333 AK Leiden, The Netherlands; Department of Psychiatry, Leiden University Medical Center, Leiden, The Netherlands; Department of Psychiatry/EMGO Institute for Health and Care Research, VU University Medical Center, Amsterdam, The Netherlands

**Keywords:** Experiential avoidance, Rumination, Worry, Neuroticism, Depressive disorder

## Abstract

Our study aim was to investigate how experiential avoidance ‘works together’ with bordering psychological constructs (i.e., rumination, worry and neuroticism) in predicting the onset, relapse and maintenance of depressive disorders. We performed a longitudinal cohort study with repeated assessments after 2 and 4 years in a sample of 737 persons with a 6-month recency dysthymic and/or major depressive disorder, a sample of 1150 remitted persons with a history of previous depressive disorders; and a sample of 626 persons with no 6-month recency depressive or anxiety disorders and no previous depressive disorders. Experiential avoidance predicted onset, relapse as well as maintenance of depressive disorders during the 4-year follow-up period. However, after controlling for rumination, worry and neuroticism, experiential avoidance no longer significantly predicted onset, relapse or maintenance of depressive disorders in contrast to repetitive thinking in the form of rumination or worry. Experiential avoidance also did not mediate or moderate the effect of rumination, worry and neuroticism.

## Introduction

Experiential avoidance is described as consisting of two related parts: (a) the unwillingness to remain in contact with aversive private experience (including bodily sensations, emotions, thoughts, memories, and behavioral predispositions), and (b) action taken to alter the aversive experiences or the events that elicit them (Hayes et al. 1996). Experiential avoidance has been hypothesized to play an important role in the etiology, maintenance and modification of various forms of psychopathology (Hayes et al. 2004), anxiety and depression in particular (for reviews see Chawla and Ostafin 2007; Hayes et al. 1996). Based on current literature experiential avoidance is considered to constitute a transdiagnostic risk factor as it leads to multiple disorders (Baer 2007; Barlow et al. 2004; Harvey et al. 2004).

Experiential avoidance has been associated with bordering psychological constructs associated with depression such as rumination, worry and neuroticism based on the presupposition that these constructs are related to experiential avoidance and that experiential avoidance could mediate or moderate the effect of these constructs on depressive disorder. As the history of psychology is replete with constructs that have difficulty establishing incremental validity above and beyond those that came before (Haynes and Lench 2003; Smith et al. 2003), it is crucial to study into more detail how experiential avoidance works together with other psychological constructs in predicting depression. The present longitudinal study aims to investigate to what extent experiential avoidance in relation to bordering psychological constructs (i.e., rumination, worry and neuroticism) predicts onset, relapse and maintenance of depressive disorder.

Cross-sectional studies in non-clinical samples suggest that experiential avoidance is moderately to strongly associated with symptoms of depression (Bjornsson et al. 2010; Cribb et al. 2006; Morina 2011; Tull and Gratz 2008) and that this association remains after controlling for shared variance with anxiety symptoms (Cribb et al. 2006; Tull and Gratz 2008). Although experiential avoidance represents a generalized psychological risk factor for depressive disorders, there have been few studies investigating the association of experiential avoidance with other higher-order personality traits associated with depressive disorders such as neuroticism (Kotov et al 2010). Available evidence indicates that although experiential avoidance is associated with negative emotionality/neuroticism (Boelen and Reijntjes 2008; Bond and Bunce 2003; Hayes et al. 2004; Kashdan et al. 2006), experiential avoidance can be distinguished from neuroticism as experiential avoidance remained significantly associated with self-reported symptoms of depression after controlling for neuroticism (Boelen and Reijntjes 2008).

Given its avoidant function, slightly paradoxically, experiential avoidance has also been associated with rumination. Although rumination is associated with a cognitive focus on negative thoughts, avoidance of the affect associated with the ruminative content may, similarly to worry, be an avoidant property of rumination (Moulds et al 2007). Rumination may act as a ‘smokescreen’ enabling individuals to suppress or disconnect from negative emotions. In line with this presupposition, cross-sectional studies in non-clinical samples showed that experiential avoidance is positively associated with rumination (Cribb et al. 2006; Giorgio et al. 2010; Morin 2011). Moreover, experiential avoidance has been found to mediate the association of the anxiety sensitivity dimensions of fear of cognitive dyscontrol and fear of publicly observable anxiety reactions with depressive symptom severity in a cross-sectional study (Tull and Gratz 2008) and the association of rumination during bereavement with depressive symptom severity in a longitudinal study (Eisma et al. 2013). In addition, Bjornsson et al. (2010) reported an interaction effect between experiential avoidance and rumination on the basis of cross-sectional data, suggesting that experiential avoidance is only associated with depressive symptoms when rumination is high. However, they failed to replicate this moderation effect in their longitudinal analyses. Moreover, the interaction of rumination and experiential avoidance also did not predict depressive symptoms over and above the main effect of both variables in widowed mothers (Morina 2011).

Based on Borkovec (1994) seminal theory ascribing an avoidant function to worry, experiential avoidance is also hypothesized to be related to worry. In line, cross-sectional studies in non-clinical samples have found a significant and positive relationship of experiential avoidance with pathological worry (Roemer et al. 2005; Ruiz 2014a, b; Santanello and Gardner 2007). However, experiential avoidance in relation to worry has mainly been investigated in the context of anxiety (e.g., Roemer et al. 2005; Santanello and Gardner 2007), although worry may also be a psychological risk factor for depressive disorders (Olatunji et al. 2010). Some studies even found that patients with generalized anxiety disorder and major depression did not differ in the frequency and intensity of worry (e.g., Starcevic 1995; Wells and Carter 2009).

The studies reviewed above are compatible with experiential avoidance being an independent, overlapping, proxy, mediating or moderating factor in depression. Of note is that most of the studies are cross-sectional using non-clinical samples. A fundamental limitation of cross-sectional studies is that such a design precludes concluding whether psychological constructs are risk factors (i.e., a correlate shown to precede outcome) or can be better seen as correlates, signs and symptoms, concomitants or even consequences. Moreover, none of the available longitudinal studies examined presence of depressive disorders instead of depressive symptom severity as outcome variable. An exception is our previous longitudinal study (Spinhoven et al. 2014) showing that experiential avoidance may be conceptualized as a transdiagnostic factor affecting the course and development of comorbidity of distress and fear disorders. However, this study did not examine the specific relationship of experiential avoidance with onset, relapse and maintenance of depressive disorders and also did not consider the predictive role of experiential avoidance in relationship to bordering psychological constructs.

The primary aim of the present *longitudinal* study is to investigate in an explorative way how experiential avoidance works together with other psychological constructs (i.e., rumination, worry and neuroticism) in predicting onset, relapse and maintenance of depressive *disorders*. In line with the approach of Kraemer et al. (2001), we will differentiate between five different and clinically important ways in which experiential avoidance as a putative risk factor may work together with other psychological constructs to influence future outcome: as an independent, overlapping, proxy, mediating or moderating risk factor. More specifically, our first aim is to investigate whether experiential avoidance can best be conceptualized as an independent risk factor (i.e. experiential avoidance is not correlated with another psychological construct and both predict future outcome), an overlapping risk factor (i.e., experiential avoidance is correlated with another psychological construct and both independently predict outcome), or a proxy risk factor (i.e. experiential avoidance is correlated with another psychological construct and when that relationship is taken into account does not uniquely predict outcome).

Our second and third study aims were to investigate experiential avoidance as a moderator (i.e., experiential avoidance explains under what conditions other psychological constructs predict onset, relapse or maintenance of depressive disorders) (aim 2) and as a mediator (i.e., experiential avoidance explains how another psychological construct predicts future depressive disorders) (aim 3). For these aims, we will use the MacArthur approach (Kraemer et al. 2001, 2008), which defines strict eligibility criteria to identify whether a variable is a candidate for consideration as a potential moderator (or mediator) based on association and temporal precedence. More specifically this approach stipulates (a) that experiential avoidance as a moderator must precede another psychological construct as focal predictor and that moderator and predictor are independent (moderation) and (b) that another psychological construct as a predictor must precede experiential avoidance as a mediator and that predictor and mediator are associated (mediation). The present longitudinal study provides a unique opportunity to study moderation and mediation in this way, because this approach requires at least three time points to establish moderation or mediation.

## Methods

### Sample

The Netherlands Study of Depression and Anxiety (NESDA) is an ongoing cohort study designed to investigate determinants, course and consequences of depressive and anxiety disorders. The NESDA sample of 2981 adults (18–65 years) includes participants with a lifetime and/or current anxiety and/or depressive disorder (n = 2329; 78 %) and healthy controls (persons without depressive or anxiety disorders; n = 652; 22 %). To include various developmental stages of disorders and different levels of severity, participants were recruited from general practices (n = 1610; 54 %), mental health organizations (n = 807; 27 %), and the general population (n = 564; 19 %). Community-based subjects with depressive or anxiety disorders were previously identified in two population-based studies: Nemesis (Bijl et al. 1998) and Ariadne (Landman-Peeters et al. 2005). Primary care patients were identified through a 3-stage screening procedure (involving the K10 and the CIDI short form) among patients of 65 General Practitioners consulting for any reason in a 4-month period. In secondary care, patients were recruited when newly enrolled for a depressive or anxiety disorder at one of the 17 participating mental health organization locations. General exclusion criteria were a primary diagnosis of severe psychiatric disorders such as psychotic, obsessive compulsive, bipolar or severe addiction disorder, and not being fluent in Dutch. A detailed description of the NESDA design and sampling procedures has been given elsewhere (Penninx et al. 2008). The research protocol was approved by the Ethical Committees of the participating universities and all respondents provided written informed consent.

The baseline assessment included demographic and personal characteristics, a standardized diagnostic psychiatric interview, an extensive set of psychological measures and a medical assessment including blood sampling. After 2 (T2), 4 (T4), and 6 years (T6) a face-to-face follow-up assessment was conducted with a response of 87.1 % (n = 2596) at T2, of 80.6 % (n = 2402) at T4 and of 75.7 % (n = 2256) at T6. Experiential avoidance was measured at T2 for the first time and consequently the T2 measurement constituted the baseline measurement in the present study.

For the purpose of the present study we selected the following three subgroups: (a) persons with a 6-month recency dysthymic and/or major depressive disorder at T2, that could persist during the follow-up (n = 626; depressed group); (b) persons with a history of previous depressive disorders but no 6-month recency dysthymic or major depressive disorder at T2, who could have a relapse during the follow-up (n = 1150; previously depressed group); and (c) persons with no history of previous depressive disorders and no 6-month recency dysthymic or major depressive disorder or any other emotional disorder at T2, who could develop depression during the follow-up (n = 737; healthy group).

### Measures

#### Psychiatric Diagnosis

Past and present 6-month recency DSM-IV (APA 1994) depressive [Major Depressive Disorder (MDD), Dysthymia (DYS)] and anxiety [Panic Disorder with or without Agoraphobia (PD), Social Anxiety Disorder (SAD), Generalized Anxiety Disorder (GAD), Agoraphobia without panic (AGO)] disorders at T2, T4 and T6 were assessed using the Composite Interview Diagnostic Instrument (CIDI, version 2.1). The CIDI is a fully standardized diagnostic interview, that is used worldwide to classify psychiatric diagnoses according to DSM-IV criteria (APA 1994). It has shown high interrater reliability, high test–retest reliability and high validity for depressive and anxiety disorders (Wittchen 1994). The CIDI was administered in NESDA by fully trained research assistants, including psychologists, nurses or residents in psychiatry. Maintenance was defined as a dysthymic and/or major depressive disorder between T2 and T6 in persons with a 6-month recency depressive disorder at T2. Relapse was defined as a dysthymic and/or major depressive disorder between T2 and T6 in persons with no 6-month recency depressive disorder at T2, but with a history of previous depressive disorders. Onset was defined as a dysthymic and/or major depressive disorder between T2 and T6 in persons with no 6-month recency depressive or anxiety disorder at T2 and in addition no history of previous depressive disorders.

#### Depression Severity

Severity of depressive symptoms at T2 and T4 was measured with the Dutch version of the 30-item *Inventory of Depressive Symptomatology* self-report version (IDS-SR; Rush et al. 1986), which has shown high correlations with observer-rated scales such as the Hamilton Depression Scale (Rush et al. 1996). Internal consistency of the IDS-SR in the present study was .89 at T2 and .90 at T4.

#### Experiential Avoidance

Experiential avoidance at T2 and T4 was assessed using the Dutch version of the 9-item Acceptance and Action Questionnaire-I (AAQ-I; Hayes et al. 2004). Items are scored on a 7-point Likert scale ranging from ‘1 = never true’ to ‘7 = always true’ (e.g.: ‘I rarely worry about getting my anxieties, worries, and feelings under control’). A previous study of the Dutch AAQ-I showed that a one-factor model, with AAQ items constituting a single dimension of experiential avoidance, fitted the data well. Also the internal consistency (.74) and temporal stability of the AAQ (.82) were satisfactory (Boelen and Reijntjes 2008). Internal consistency of the AAQ-I in the present study was .69 at T2 and .74 at T4. The correlation between both measurements was .70.

#### Worry

Worry at T2 and T4 was measured with the Dutch version of the Penn State Worry Questionnaire (PSWQ; Meyer et al. 1990; Van Rijsoort et al. 1999). This questionnaire consists of 16 items rated on a 5-point Likert scale ranging from ‘1 = not at all typical of me’ to ‘5 = very typical of me’ (e.g.: ‘Once I start worrying, I cannot stop’). The PSWQ consists of two subscales: a ‘General worry’ subscale (11 items) and a ‘Not-worry’ subscale (5 items) (Van Rijsoort et al. 1999). The ‘General worry’ subscale accounts for most of the variance in PSWQ scores (Meyer et al. 1990; Van Rijsoort et al. 1999), and only this subscale was administered in the NESDA study. The PSWQ has been proven to be a valid measure of trait worrying unaffected by the content of the worry (Davey 1993) with high internal consistency, good test–retest reliability and unaffected by social desirability (Meyer et al. 1990). Internal consistency of the General worry scale in the present study was .96 at T2 and .96 at T4.

#### Rumination

Rumination at T2 and T4 was assessed using the subscale Rumination on Sadness of the revised version of the Leiden Index of Depression Sensitivity (LEIDS-R; Van der Does 2002; Williams et al. 2008). The subscale rumination on sadness (RUM) consists of six items. Participants are asked to indicate whether and how their thinking patterns change when they experience mild dysphoria by scoring each item on a 5-point Likert-scale ranging from 0 ‘not at all’ to 4 ‘very strongly’ applicable to me (e.g.: ‘When I feel sad, I spend more time thinking about the possible causes of my moods’). LEIDS-RUM scores are significantly associated with scores for rumination on the Ruminative Response Scale (RRS): .51 after controlling for current depressive symptoms, showing that the observed relationship is independent of current mood state (Moulds et al. 2008). In the present sample the internal consistency of the RUM-scale was .84 at T2 and .85 at T4.

#### Neuroticism

Neuroticism at T2 and T4 was measured with the subscale for neuroticism of the Dutch version of the 60-item NEO Five-Factor Inventory (NEO-FFI; Costa and McCrae 1992, 1995; Dutch version Hoekstra et al. 1996). The NEO-FFI questionnaire measures the following five personality domains: Neuroticism, Extraversion, Agreeableness, Conscientiousness and Openness to Experience. Internal consistency values range from .74 to .89. Cronbach’s alpha’s of the neuroticism subscale in the present study was .81 at T2 and .81 at T4.

### Statistical Analyses

As we pursued to assess experiential avoidance as an independent, overlapping, proxy, moderating and mediating risk factor for onset, relapse and maintenance of depressive disorders, all statistical analyses described below were conducted separately in the healthy, previously depressed and depressed group.

First, univariable logistic regression analyses were conducted to assess the predictive value of T2 sociodemographic (i.e., age, gender, and education), clinical [i.e., history of previous depressive episodes, depressive symptom severity and co-morbid anxiety disorders (GAD, SAD, PD, and AGO)], and psychological variables (i.e., experiential avoidance, rumination, worry, and neuroticism) for presence of depressive disorders during the follow-up period between T2 and T6. Next, two multivariable models were constructed, one model including all sociodemographic and clinical variables (Model 1A) and one model including all psychological variables (Model 1B). Next, in order to determine which variables were independent risk factors for presence of depressive disorders during the follow-up period, all statistically significant variables in the two multivariable models were entered into a final multivariable model (Model 2). By definition history of previous depressive disorders and comorbid anxiety disorders were not included as covariates in the healthy group.

Next, in accordance with the requirements of the MacArthur approach, we first assessed whether experiential avoidance at T2 was significantly associated with psychological constructs at T4 (i.e. rumination, worry and neuroticism) in order to examine whether experiential avoidance was eligible as a putative moderator. In the absence of significant associations, we planned to execute logistic regression analyses to test the hypothesis that T2 experiential avoidance would moderate the relationship of the other psychological constructs at T4 with presence of depressive disorders during T4 and T6 controlling for presence of depressive disorders during T2 and T4.

Finally, mediation analyses were conducted using the bootstrapping procedure for multiple mediators for SPSS as developed by Preacher and Hayes (2008). Bootstrapping is a nonparametric resampling method that generates an empirical approximation of the sampling distribution of a statistic from the data and, as such, avoids the power problems associated with non-normality in the sampling distribution. The procedure provides point estimates and 95 % CI for the total and individual indirect effects. We used 5000 bootstrap resamples and focused on the bias corrected 95 % CI (95 % BCI) with point estimates of indirect effects being considered significant if zero is not included in the interval. In separate analyses we tested whether each of the psychological constructs at T2 had a significant effect on presence of depressive disorders during T4 and T6 through T2–T4 changes in experiential avoidance, while controlling for presence of depressive disorders during T2–T4. For these analyses we calculated T4 residualized gain scores for experiential avoidance, which are uncorrelated to corresponding T2 scores in trying to meet the criterion of temporal precedence of predictor variables in mediation analysis. Using the formula of MacKinnon et al. (2007) the effect size of the indirect effects was assessed by calculating the proportion of the effect of the predictor variable on the dependent variable that is accounted for by the mediator (i.e., 1−c′/c).

Statistical analyses were run using SPSS version 21 (IBM Corp. 2012) and a significance level of *p* < .05 was used for all analyses.

## Results

### Sample Characteristics

In our total sample of 2513 participants, 626 (24. %) had a six-month recency MDD and/or DYS and 1678 (66.8 %) a lifetime MDD and/or DYS at T2. Of the 241 persons with a six-month recency DYS at T2, 188 had concurrent MDD (78.0 %) and of the 663 persons with a lifetime DYS at T2 615 (92.8 %) had a life time MDD. Study dropouts (n = 406; 16.2 %) between T2 and T6 did not differ from study completers (n = 2107; 83.8 %) regarding gender distribution, level of rumination, prevalence of GAD and AGO and history of previous depressive episodes. However, in comparison to completers dropouts were significantly older and less educated and showed higher levels of depressive symptoms, experiential avoidance, worry and neuroticism as well as higher prevalence rates of DYS/MDD, SAD and PD. However, the effect sizes for the differences between both groups were negligible (Cohens’*d* < .2 or Cramer’s phi < .10).

 Table 1 shows sociodemographic, clinical and psychological characteristics of our three subgroups at T2: (a) persons with a 6-month recency dysthymic and/or major depressive disorder at T2 (n = 626; depressed group); (b) persons with no 6-month recency dysthymic or major depressive disorder at T2, but with a history of previous depressive disorders (n = 1150; previously depressed group); and (c) persons with no 6-month recency dysthymic or major depressive disorder or any other emotional disorder at T2 and also no history of previous depressive disorders (n = 737; healthy group). As can be derived from this table, level of depression, experiential avoidance, rumination, worry and neuroticism significantly differed between groups with depressed participants scoring higher than previously depressed participants, who scored higher than healthy controls on each of these variables.

**Table 1:** Overview of sociodemographic, clinical and psychological characteristics of the three subgroups^a^

	1. Depressed group (n = 626)		2. Previously depressed group (n = 1150)		3. Healthy group (n = 737)		Overall statistics χ^2^ (*df*)/F (*df*) *p* value	Contrasts
	M	SD	M	SD	M	SD		
Age	44.8	12.1	43.9	12.7	43.6	14.5	1.64 (2) ns	
Female gender (n/%)	419	66.9	790	68.7	451	61.2	.58 (2) ns	
Years of education	11.9	3.4	12.4	3.3	13.1	3.2	16.48 (2)*	1 = 2<3
Previous depression (n/%)	268	42.8	517	45.0	0	0	.76 (2) ns	
IDS-SR	28.0	12.1	14.7	9.5	7.5	6.4	775.12 (2)*	1 > 2>3
GAD (n/%)	141	22.5	45	3.9	0	0	149.74 (1)*	1 > 2
SAD (n/%)	192	30.7	132	11.5	0	0	100.11 (1)*	1 > 2
PD (n/%)	169	27.0	89	7.7	0	0	121.07 (1)*	1 > 2
AGO (n/%)	63	10.1	65	5.7	0	0		1 > 2
AAQ-I	38.2	6.6	32.8	7.0	27.8	6.4	215.82 (2)*	1 > 2> 3
LEIDS:RUM	12.0	4.6	8.4	4.7	4.5	3.8	432.47 (2)*	1 > 2> 3
PSWQ	37.0	9.9	28.5	10.8	20.0	8.6	439.73 (2)*	1 > 2> 3
NEO-FFI:N	41.1	7.0	33.7	7.7	26.8	7.1	611.70 (2)*	1 > 2> 3

*IDS-SR* inventory of depressive symptomatology–self-report, *GAD* generalized anxiety disorder, *SAD* social anxiety disorder, *PD* panic disorder, *AGO* agoraphobia, *AAQ-I* acceptance and action questionnaire-I, *LEIDS:RUM* Leiden index of depression sensitivity-revised: rumination on sadness subscale, *PSWQ* Penn State worry questionnaire, *NEO-FF:*N NEO five-factor inventory: neuroticism subscale, *ns* non-significant

* *p* < .001

^a^Eighty-nine participants who completed the T6 assessment did not participate in the T4 assessment

### Prediction of Depressive Disorders During T2–T6 by Psychological Constructs at T2

Table 2 shows the correlations among the psychological constructs and severity of depressive symptoms at T2. As can be derived from the lower triangular of this table, all psychological constructs are significantly intercorrelated with a moderate effect size (.50 ≤ r < .80). Experiential avoidance was significantly associated with neuroticism (r = .68, *p* < .001), worry (r = .64, *p* < .001), rumination (r = .55, *p* < .001), and severity of depressive symptoms (r = .58, *p* < .001).

**Table 2:** Correlations of psychological constructs and symptom severity

Variables	1	2	3	4	5
1. AAQ-I	–	.47	.56	.63	.53
2. LEIDS:RUM	.55	–	.66	.66	.58
3. PSWQ	.64	.64	–	.78	.70
4. NEO-FFI: N	.68	.62	.76	–	.69
5. IDS-SR	.58	.55	.66	.74	–

All *p* < .001

*AAQ-I* acceptance and action questionnaire-I, *LEIDS:RUM* Leiden index of depression sensitivity-revised: rumination on sadness subscale, *PSWQ* Penn State worry questionnaire, *NEO-FFI:N* NEO five-factor inventory: neuroticism subscale, *IDS-SR* inventory of depressive symptomatology—self-report; Correlations in lower triangular represent correlation among T2 measurements; Correlations in upper triangular represent correlations of AAQ-I at T2 with other variables at T4 and intercorrelations at T4 among the other variables

For 2147 of the 2513 persons (85.4 %) sufficient T4 and/or T6 data were available to determine onset, relapse or maintenance of depressive disorders. In the healthy group (n = 635; attrition = 13.8 %) the incidence of depression was 9.0 %, in the previously depressed group (n = 977; attrition = 15.0 %) the relapse rate was 36.9 % and 75.7 % of the persons in the depressed group (n = 535; attrition = 14.5 %) had depressive disorder during the follow-up period. Univariable logistic regression analyses showed that experiential avoidance at T2 predicted onset of depressive disorders during T2–T6 (OR 1.89; 95 % CI 1.34–2.66; see Table 3), relapse of depressive disorders during T2–T6 (OR 1.72; 95 % CI 1.47–2.03; see Table 4), and maintenance of depressive disorders during T2–T6 (OR 2.15; 95 % CI 1.66–2.78; see Table 5). However, after controlling for other psychological constructs (i.e., worry, rumination, and neuroticism), experiential avoidance was no longer predictive of onset of depressive disorders (OR 1.23; 95 % CI .81–1.87; see multivariable Model 1b in Table 3), relapse of depressive disorders (OR 1.10; 95 % CI .90–1.36; see multivariable Model 1b in Table 4), and maintenance of depressive disorders (OR 1.35; 95 % CI .97–1.88; see multivariable Model 1b in Table 5).

**Table 3:** Sociodemographic, clincal and psychological predictors of T2–T6 depressive disorders in the healthy group with follow-up data (n = 635)

	Univariable model 1^a^		Multivariable model 1^b^		Multivariable model 2^c^	
	OR	95 % CI	OR	95 % CI	OR	95 % CI
*Model 1A*						
Sociodemographic characteristics						
Age	.99	.97–101				
Gender	1.84	1.00–3.39				
Education	.95	.87–1.04				
Clinical characteristics						
IDS-SR (per SD increase)	**4.73**	**3.05–7.35**	**4.73**	**3.05–7.35**	**3.78**	**2.35–6.11**
*Model 1B*						
Psychological constructs						
AAQ-I (per SD increase)	**1.89**	**1.34–2.66**	1.23	.81–1.87		
LEIDS:RUM (per SD increase)	**2.11**	**1.51–2.96**	**1.55**	**1.03–2.34**	**1.51**	**1.03–2.21**
PSWQ (per SD increase)	**2.00**	**1.46–2.75**	1.15	.71–1.85		
NEO-FFI:N (per SD increase)	**2.39**	**1.68–3.83**	**1.51**	**.91–2.49**		

The bold values indicate statistical significance (*p* < .05)

*IDS-SR* inventory of depressive symptomatology-self-report, *AAQ-I* acceptance and action questionnaire-I, *LEIDS:RUM* Leiden index of depression sensitivity-revised: rumination on sadness subscale, *PSWQ* Penn State worry questionnaire, *NEO-FFI:N* NEO five-factor inventory: neuroticism subscale

^a^Based on univariable logistic regression

^b^Based on multivariable logistic regression with all sociodemographic and clinical variables (Model 1A) or all psychological variables (Model 1B) in model

^c^Based on multivariable logistic regression with all variables entered in model that had *p* < .05 in Model 1A or 1B

**Table 4:** Sociodemographic, clinical and psychological predictors of T2–T6 depressive disorders in the previously depressed group with follow-up data (n = 977)

	Univariable model 1^a^		Multivariable model 1^b^		Multivariable model 2^c^	
	OR	95 % CI	OR	95 % CI	OR	95 % CI
*Model 1A*						
Sociodemographic characteristics						
Age	.99	.98–1.00				
Gender	1.26	.95–1.68				
Education	**.94**	**.90–.98**	.97	.93–1.02		
Clinical characteristics						
Previous depression	.89	.68–1.16				
IDS-SR (per SD increase)	**2.36**	**1.96–2.84**	**2.18**	**1.79–2.66**	**1.81**	**1.45–2.27**
GAD	**2.16**	**1.14–4.08**	1.05	.53–2.10		
SAD	**2.32**	**1.54–3.49**	1.47	.94–2.31		
PD	1.46	.91–2.34				
AGO	**2.16**	**1.23–3.76**	1.61	.88–2.94		
*Model 1B*						
Psychological constructs						
AAQ-I (per SD increase)	**1.72**	**1.47–2.03**	1.10	.90–1.36		
LEIDS:RUM (per SD increase)	**1.83**	**1.56–2.15**	**1.34**	**1.11–1.62**	**1.38**	**1.15–1.66**
PSWQ (per SD increase)	**2.04**	**1.74–2.39**	**1.46**	**1.18–1.82**	**1.35**	**1.10–1.66**
NEO-FFI:N (per SD increase)	**1.98**	**1.66–2.35**	1.28	.99–1.63		

The bold values indicate statistical significance (*p* < .05)

*IDS-SR* inventory of depressive symptomatology-self-report, *GAD* generalized anxiety disorder, *SAD* social anxiety disorder, *PD* panic disorder, *AGO* agoraphobia, *AAQ-I* acceptance and action questionnaire-I, *LEIDS:RUM* Leiden index of depression sensitivity-revised: rumination on sadness subscale, *PSWQ* Penn State worry questionnaire, *NEO-FFI:N* NEO five-factor inventory: neuroticism subscale

^a^Based on univariable logistic regression

^b^Based on multivariable logistic regression with all sociodemographic and clinical variables (Model 1A) or all psychological variables (Model 1B) in model

^c^Based on multivariable logistic regression with all variables entered in model that had *p* < .05 in Model 1A or 1B

**Table 5:** Sociodemographic, clincal and psychological predictors of T2–T6 depressive disorders in the depressed group with follow-up data (n = 535)

	Univariable model^a^		Multivariable model 1^b^		Multivariable model 2^c^	
	OR	95 % CI	OR	95 % CI	OR	95 % CI
*Model 1A*						
Sociodemographic characteristics						
Age	.97	.97–1.00				
Gender	.81	.53–1.24				
Education	.99	.94–1.06				
Clinical characteristics						
Previous depression	1.27	.85–1.91				
IDS-SR (per SD increase)	**2.03**	**1.61–2.56**	**2.00**	**1.51–2.39**	**1.52**	**1.18–1.96**
GAD	1.57	.95–2.57				
SAD	**2.10**	**1.30–3.40**	**1.69**	**1.02–2.79**	1.33	.78–2.29
PD	1.55	.96–2.49				
AGO	1.05	.53–2.07				
*Model 1B*						
Psychological constructs						
AAQ-I (per SD increase)	**2.15**	**1.66–2.78**	1.35	.97–1.88		
LEIDS:RUM (per SD increase)	**1.75**	**1.36–2.24**	1.14	.85–1.53		
PSWQ (per SD increase)	**2.51**	**1.90–3.30**	**1.69**	**1.17–2.44**	**2.04**	**1.52–2.75**
NEO-FFI:N (per SD increase)	**2.32**	**1.76–3.07**	1.29	.88–1.89		

The bold values indicate statistical significance (*p* < .05)

*IDS-SR* inventory of depressive symptomatology-self-report, *GAD* generalized anxiety disorder, *SAD* social anxiety disorder, *PD* panic disorder, *AGO* agoraphobia, *AAQ-I* acceptance and action questionnaire-I, *LEIDS:RUM* Leiden index of depression sensitivity-revised: rumination on sadness subscale, *PSWQ* Penn State worry questionnaire, *NEO-FFI:N* NEO five-factor inventory: neuroticism subscale

^a^Based on univariable logistic regression

^b^Based on multivariable logistic regression with all sociodemographic and clinical variables (Model 1A) or all psychological variables (Model 1B) in model

^c^Based on multivariable logistic regression with all variables entered in model that had *p* < .05 in Model 1A or 1B

After additionally controlling for significant clinical characteristics as derived from multivariable Model 1A, onset of depressive disorders was predicted by symptom severity (OR 3.78; 95 % CI 2.35–6.11) and rumination (OR 1.51; 95 % CI 1.03–2.21) (see multivariable Model 2 in Table 3); relapse of depressive disorders by symptom severity (OR 1.81; 95 % CI 1.45–2.27), worry (OR 1.35; 95 % CI 1.10–1.66), and rumination (OR 1.38; 95 % CI 1.15–1.66) (see multivariable Model 2 in Table 4); and maintenance of depressive disorders by symptom severity (OR 1.52; 95 % CI 1.18–1.96), and worry (OR 2.04; 95 % CI 1.52–2.75) (see multivariable Model 2 in Table 5).

### Experiential Avoidance at T2 as a Moderating Variable

Table 2 shows the correlations of experiential avoidance at T2 with the other psychological constructs and severity of depressive symptoms at T4. As can be derived from the upper triangular of this table, experiential avoidance was significantly associated with neuroticism (r = .63, *p* < .001), worry (r = .56, *p* < .001), rumination (r = .47, *p* < .001) and severity of depressive symptoms (r = .53, *p* < .001). These moderately strong associations indicate that according to the MacArthur approach experiential avoidance at T2 is not eligible as a putative moderator of the predictive value of T4 worry, rumination, and neuroticism for presence of depressive disorders during T4–T6.

### Experiential Avoidance at T4 as a Mediating Variable

Table 6 shows the associations of worry, rumination, and neuroticism at T2 with changes in experiential avoidance from T2 to T4 (a-paths in the mediation model). All relationships except for rumination with changes in experiential avoidance in the depressed and previously depressed group were statistically significant with correlations varying from negligible in size (PSWQ with changes in experiential avoidance in the depressed group: r = .06, *p* < .05) to small (neuroticism with changes in experiential avoidance in the healthy group: r = .25, *p* < .001). Subsequent mediation analyses showed that only the effect of T2 neuroticism in the depressed group on presence of depressive disorders during T4–T6 through T2–T4 changes in experiential avoidance while controlling for depressive disorders during T2–T4 was statistically significant (estimate = .008; 95 % BCI .002–017). The effect size of .06 however indicates that only a small proportion of the predictive effect of neuroticism on maintenance of depressive disorders may be attributable to T2–T4 changes in experiential avoidance. Note that without correcting T4 scores of experiential avoidance for corresponding T2 scores, the indirect effect would have been much larger (estimate = .04; 95 % BCI .021–.058) with an effect size of .44. However, this effect mainly reflects the stable association of T2 neuroticism with T4 experiential avoidance (*β* = .50, *p* < .001) and not the effect of neuroticism on subsequent changes in experiential avoidance.

**Table 6:** Estimates of mediation analyses predicting T4–T6 depressive diagnoses by psychological constructs at T2 through T2–T4 changes on AAQ-I controlling for T2–T4 depressive disorders based on 5000 bootstrap resamples

Predictor	Total effect (c)	SE	Direct effect (c′)	SE	a-Path	SE	b-Path	SE	Indirect effect (Σ a × b)	SE	Effect size mediation (1−c′/c)
*Depressed group*											
PSWQ	.05***	.01	.05***	.01	.06*	.03	.07***	.02	.00	.01	.05
LEIDS:RUM	.09***	.02	.09***	.02	.05	.06	.07***	.02	.00	.00	.02
NEO-FFI:N	.08***	.02	.08***	.02	.13**	.04	.06**	.02	.01*	.00	.06
*Previously depressed group*											
PSWQ	.04***	.01	.04***	.01	.07***	.02	.03	.02	.00	.00	.04
LEIDS:RUM	.09***	.02	.09***	.02	.07	.04	.03	.02	.00	.00	.02
NEO-FFI:N	.05***	.01	.04***	.01	.18***	.03	.02	.02	.00	.00	.08
*Healthy group*											
PSWQ	.05*	.02	.05*	.02	.16***	.03	.00	.04	.00	.01	.00
LEIDS:RUM	.08	.05	.08	.05	.18**	.06	.01	.04	.00	.01	.02
NEO:FFI	.04	.03	.04	.03	.25***	.03	.00	.04	.00	.01	.02

The bold values indicate statistical significance (*p* < .05)

*AAQ-I* acceptance and action questionnaire-I, *LEIDS:RUM* Leiden index of depression sensitivity-revised: rumination on sadness subscale, *PSWQ* Penn State worry questionnaire, *NEO-FFI:N* NEO five-factor inventory: neuroticism subscale

*** *p* < .001; ** *p* < .01; * *p* < .05

## Discussion

Our first research question was whether experiential avoidance is an independent, overlapping or proxy risk factor of onset, relapse, or maintenance of depressive disorders. In accordance with previous studies experiential avoidance was significantly associated with rumination (Cribb et al. 2006; Giorgio et al. 2010; Morina 2011), worry (Roemer et al. 2005; Ruiz 2014a, b; Santanello and Gardner 2007), and neuroticism (Boelen and Reijntjes 2008; Bond and Bunce 2003; Hayes et al. 2004; Kashdan et al. 2006). These moderately strong interrelationships among psychological constructs indicate that experiential avoidance cannot be considered to constitute an independent risk factor and that the predictive value of experiential avoidance for depressive disorders has to be considered together with associated psychological constructs.

Next we examined how experiential avoidance worked together with bordering psychological constructs (i.e. rumination, worry, and neuroticism) as an overlapping or proxy risk factor of depressive disorders. Assessed separately experiential avoidance at T2 predicted onset, relapse and maintenance of depressive disorders during the 4-year follow-up period. However, after controlling for the other psychological constructs at T2, experiential avoidance no longer significantly predicted neither onset, nor relapse or maintenance of depressive disorders. After additionally controlling for significant clinical risk factors, onset of depressive disorders was predicted by symptom severity and rumination, relapse of depressive disorders by symptom severity, rumination and worry and maintenance of depressive disorders by symptom severity and worry. These results suggest that in the context of depressive disorders, experiential avoidance can best be conceptualized as a proxy risk factor for repetitive negative thinking in the form of rumination and worry constituting more dominant risk factors for depressive disorders (Olatunji et al. 2013). Conceivably experiential avoidance will show a unique relationship with other emotional disorders than depression as the psychological constructs investigated were highly interrelated and the relative strength of their relationships with particular disorders may depend on the type of disorder investigated. The high intercorrelations among the psychological constructs make it hard to draw strong conclusions on whether experiential avoidance can best be regarded as a proxy or overlapping risk factor.

Our second research question was to examine whether experiential avoidance is part of a possible causal chain in which experiential avoidance as a moderator explains how or why another psychological construct as a focal predictor affects future outcome. The widely used definition of a moderator as specifying on whom or under what conditions another variable will operate to produce an outcome (Baron and Kenny 1986) may be too imprecise to examine this question as on the basis of this definition almost any two risk factors, given a judicious choice of linear model and a large enough sample, could be found to simultaneously moderate each other (Kraemer et al. 2001). As already suggested by Baron and Kenny (1986) moderation models may be most appropriate in case of interaction between two relatively independent factors (Baron and Kenny 1986). In line, Nolen-Hoeksema and Watkins (2011) suggest to confine moderators to environmental and biological factors so as to distinguish them from psychological risk factors helping to avoid problems that emerge in interactive models involving multiple interrelated psychological risk factors. Moreover, as the Baron and Kenny approach does not specify a criterion of temporal precedence for moderation, it is unclear which of two variables is the moderator and which one is being moderated. The MacArthur approach proposes more strict eligibility criteria for a putative moderator variable and requires, besides no direct effect of moderator upon the focal predictor, temporal precedence of the moderator (Kraemer et al. 2001, 2008).

As in the present study experiential avoidance at T2 was moderately strong associated with rumination, worry and neuroticism at T4, we refrained from further examining experiential avoidance as a putative moderator on the basis of this requirement of the MacArthur approach. Not meeting the criteria of independence and temporal precedence for moderation analysis may help to explain the inconsistent results of previous studies examining experiential avoidance as a variable moderating the effect of other psychological constructs on severity levels of psychopathology. Cross-sectional studies have found stronger associations at higher levels (Bjornsson et al. 2010; Kashdan et al. 2008) or lower levels of experiential avoidance (Bardeen et al. 2013) or no relationship at all (Morina 2011; Panayiotou et al. 2014). Of note is that in these cross-sectional studies the associations of experiential avoidance with the predictor variables were all significant and on average moderately strong varying from .43 to .66. Results of longitudinal studies with two time points, in which experiential avoidance and the predictor variable were measured simultaneously and were significantly interrelated, also yielded mixed findings. Available studies show no moderation effect of experiential avoidance on rumination in predicting depressive symptoms (Bjornsson et al. 2010) or show that the association between anxiety sensitivity and anxiety becomes stronger at higher levels of experiential avoidance (Bardeen et al. 2014).

Our last research question was whether experiential avoidance is part of a possible causal chain in which experiential avoidance as a mediating variable explains how or why another variable affects future outcome (Baron and Kenny 1986; Kraemer et al. 2001). Most of the previous studies on experiential avoidance as a mediating variable are cross-sectional in nature. The use of mediation analyses with cross-sectional data, however, has been criticized on the grounds that the temporal order of variables cannot be established (Maxwell and Cole 2007). Because the present study consisted of three measurement points, we were able to perform a mediation analysis in which predictor, mediator and outcome were assessed at different time points (Kraemer et al. 2001, 2008). Nonetheless, it can be seriously questioned whether in these analyses T2 psychological constructs really preceded experiential avoidance at T4. It has been proposed that temporal order should be defined at least on the basis of when variables appear developmentally (Kraemer et al. 2001). However, regarding psychological constructs such as rumination, worry, neuroticism and experiential avoidance it is impossible to conclude that some of these constructs constitute more “fundamental” characteristics appearing earlier in development. Whether some psychological constructs can be better conceptualized as more proximal characteristics can only be tested in longitudinal studies of early childhood to adulthood. However, which of the constructs is measured first, which is entered into a regression analysis first, or which has greater predictive value does not establish temporal precedence.

Consequently, in our series of mediation analyses we tried to make the requirement of temporal precedence more stringent by analyzing T2–T4 changes in experiential avoidance as a variable mediating the effect of T2 psychological constructs on T4–T6 depressive disorders, while controlling for T2–T4 depressive disorders. Our results showed that changes in experiential avoidance failed to mediate the predictive value of worry, rumination or neuroticism on onset, relapse or maintenance of depressive disorders. We only found a marginal indirect effect of neuroticism on maintenance of depressive disorders through changes in experiential avoidance. These results question the conclusions on experiential avoidance as a mediating variable in previous cross-sectional studies in depression (e.g., Kashdan et al. 2006; Tull and Gratz 2008) and anxiety (e.g., Glick and Orsillo 2011; Kashdan et al. 2006; Ruiz 2014a; Santanello and Gardner 2007) as in cross-sectional studies temporal precedence of the predictor is absent by definition. They also question the results of longitudinal studies in which control for baseline values of the mediator is lacking. For example, the conclusion that the effect of grief rumination on depression is mediated by experiential avoidance (Eisma et al. 2013) may be premature as the association of rumination with subsequent experiential avoidance may primarily reflect the stable interrelatedness of these constructs over time. Note that these results do not imply that changes in psychological variables are not influencing the course of depressive and anxiety disorders as previous studies based on NESDA data showed that 2-year changes in experiential avoidance (Spinhoven et al. 2014) and in repetitive negative thinking (Drost et al. 2014) mediated the 4-year longitudinal association of fear disorders with distress disorders as well as the 4-year longitudinal association of distress disorders with fear disorders.

Further research of the construct of experiential avoidance seems warranted (Chawla and Ostafin 2007; Wolgast 2014). It remains unclear whether experiential avoidance should be conceptualized as a broad overarching single factor construct, or as a multifaceted construct with a number of different dimensions (e.g., cognitive, affective, and behavioral). It could even be questioned whether it is fruitful to study psychological vulnerabilities associated with depression and anxiety such as experiential avoidance in isolation, as psychological vulnerabilities for depression and anxiety are moderately to strongly correlated suggesting a common etiologic factor shared among vulnerabilities (Hong and Cheung 2014). In particular, individuals with higher levels of neuroticism have a tendency to frequently experience strong negative emotions and to evaluate these experiences as aversive. Such individuals may be more likely to engage in avoidant coping strategies (such as rumination, worry, emotion suppression, experiential avoidance, anxiety sensitivity) to manage their emotions, which paradoxically may increase the frequency/intensity of these negative emotions (Barlow et al. 2014). This high interrelatedness of psychological vulnerabilities and their common core may present a fruitful venue for transdiagnostic interventions.

Finally, self-report measures of psychological constructs may correlate poorly with actual daily processes by which experiential avoidance strategies are associated with depressive disorders. In order to determine the generalizability of studies involving self-report measures for trait-like psychological constructs, experience sampling method studies are needed. For example, Shahar and Herr (2011) showed that daily diary measurements of thought suppression, emotional suppression, rumination, distraction and lack of acceptance loaded on one single factor of experiential avoidance and that level of depression was associated with an inflexibly high level of avoidant emotion regulation. In addition, a recent diary study indicated that daily measurements of experiential avoidance were a stronger predictor of daily well-being than the AAQ, suggesting that experiential avoidance may be a context-specific regulatory strategy that might be best captured using a state-dependent measure (Machell et al. 2015).

Taken together, these results suggest that future studies on experiential avoidance should take several conceptual and methodological issues into account. Conceptually, it seems fruitful to analyze the contextual function of experiential avoidance into more detail as the relationships of various psychological and behavioral components of experiential avoidance and bordering psychological constructs may critically depend on shared functions such as avoiding aversive sensations, memories, cognitions or emotions in a particular situation. By conceptualizing experiential avoidance as a functional diagnostic dimension, many manifestations of psychopathology can be viewed as unhealthy methods of experiential avoidance (Hayes et al. 1996). Adapting such a more functional analytic approach will result in a broader perspective on psychological and behavioral components subsumed under the broader principle of experiential avoidance. Methodologically, studies using multiple ways of measuring this construct using self-report questionnaires, experience sampling methods and also experimental tasks are needed in non-clinical as well as clinical samples preferably using longitudinal designs allowing prediction of clinical outcomes and also meeting the requirement of temporal precedence for moderation and mediation analysis. The integration of different measuring perspectives could help to more clearly delineate the convergence of study findings across measurement modalities and help to evade the limitations inherent in exclusively using self-report measures.

The present study has some notable strengths: first, a longitudinal cohort study in a large representative sample of participants with various depressive and/or anxiety disorders from different recruitment settings; second, use of a structured diagnostic interview to assess presence of depressive and anxiety disorders; third, analyzing the predictive value of different psychological constructs for psychopathology concurrently; and fourth analyzing experiential avoidance as a mediating, moderating, independent, overlapping, or proxy risk factor for onset, relapse and maintenance of depressive disorders within a single study using three measurement points.

The results of this study need to be considered in light of several limitations. First, the AAQ-I as used in the present study has been criticized for having overly complex items or items showing overlap with other concepts. A new 7-item AAQ-II has been developed to improve its psychometric consistency (Bond et al. 2011). However, the correlation of the AAQ-I with the AAQ-II is very high (r = .97). Second, as the NESDA study was not specifically designed to answer the present research questions, some relevant measures of putative predictive, mediating or moderating psychological factors in depressive disorders (such as mindfulness or alternative emotion regulation strategies) were not available. Third, given the relatively high comorbidity among depressive and anxiety disorders, the differential effects of experiential avoidance and related psychological constructs on depressive disorders may have been biased by comorbid anxiety disorders, although we statistically controlled for the presence of comorbid anxiety disorders in our analyses. Fourth, although attrition was relatively low and there were only negligible differences in demographic, clinical and psychological variables between study drop-outs and study completers, attrition may restrict generalizability of our findings and may have resulted in biased estimates of associations among study variables. Fifth, in our mediation and moderation analyses we only examined linear relationships, while models including non-linear relationships or paths may be more appropriate. Sixth, the effect size of the standardized estimates of the indirect paths from psychological constructs to depressive disorders through changes in experiential avoidance as established by bootstrapping is hard to interpret as they involve the multiplication of a standardized linear regression coefficient with a standardized probit regression coefficient. This limits the possibilities to discuss the clinical relevance of our meditational study findings.

## Conclusions

Experiential avoidance, although predictive of onset, relapse and maintenance of depressive disorders in univariable analyses, did not prove to be an independent or overlapping risk factor for depressive disorders given its substantial overlap with other psychological constructs, in particular repetitive negative thinking in the form of rumination and worry. Moreover, experiential avoidance did not moderate or mediate the predictive value of the other psychological constructs for onset, relapse and maintenance of depressive disorders. Our results foremost indicate that experiential avoidance and bordering psychological constructs are highly associated and probably share a tendency to frequently experience strong negative emotions, to evaluate these experiences as aversive and to engage in avoidant coping strategies. This tendency may represent a transdiagnostic risk factor traversing a broad range of depressive and anxiety disorders. Consequently, further developing and testing of interventions targeting these psychological constructs seem warranted (Querstret and Cropley 2013) in order to investigate whether they represent causal risk factors amenable to intervention.
